# Acute Hypobaric and Hypoxic Preconditioning Reduces Myocardial Ischemia-Reperfusion Injury in Rats

**DOI:** 10.1155/2021/6617374

**Published:** 2021-03-16

**Authors:** Hirofumi Terada, Naoyuki Hirata, Yasuaki Sawashita, Sho Ohno, Yusuke Yoshikawa, Michiaki Yamakage

**Affiliations:** Department of Anesthesiology, Sapporo Medical University School of Medicine, Sapporo, Japan

## Abstract

**Background:**

Chronic and/or intermittent exposure to hypobaric hypoxia reportedly exerts cardioprotective effects against ischemia-reperfusion injury. However, few studies have focused on the cardioprotective effects of acute and/or short-term hypobaric and hypoxic exposures. This study investigated the effects of acute hypobaric hypoxia on myocardial ischemia-reperfusion injury.

**Materials and Methods:**

Rats were assigned to groups receiving normobaric normoxia (NN group), hypobaric hypoxia (HH group), or normobaric hypoxia (NH group). HH group rats were exposed to 60.8 kPa and 12.6% fraction of inspired oxygen in a hypobaric chamber for 6 h. NH group rats were exposed to hypoxic conditions under normal pressure. After each exposure, 30 min of myocardial ischemia was followed by 60 min of reperfusion. Cardiac function and infarct size were determined after reperfusion. Expression of hypoxia-inducible factor 1 alpha (HIF1*α*) was also measured.

**Results:**

Cardiac function was better preserved in the HH and NH groups than in the NN group (*p* < 0.01 each). Median infarct size/area at risk was significantly lower in the HH group (50%, interquartile range [IQR] 48–54%; *p* < 0.01 vs. NN group) and NH group (45%, IQR 36–50%; *p* < 0.01 vs. NN group) than in the NN group (72%, IQR 69–75%). HIF1*α* expression was significantly higher in the HH group (*p* < 0.05 vs. NN group) and NH group (*p* < 0.01 vs. NN group) than in the NN group.

**Conclusions:**

Exposure to acute and/or short-term hypobaric and hypoxic conditions might exert cardioprotective effects against myocardial ischemia-reperfusion injury via HIF1*α* modulation.

## 1. Introduction

The number of people exposed to high-altitude environments during activities such as mountain climbing and air travel has increased. The development of transportation technologies has granted people with easier access to such environments [1, 2]. Acute hypobaric hypoxia in a high-altitude environment can modulate internal physiological conditions and induce various clinical syndromes, including acute mountain sickness, cerebral edema, pulmonary edema, and retinal hemorrhage [3, 4]. The effects of acute hypobaric and hypoxic conditions on the cardiovascular system depend on the exposure situation (degree of barometric change and exposure duration) and the basal physical status of the individual. For instance, during the first hours of exposure to hypobaric hypoxia, hypoxic vasodilation tends to override sympathetic vasoconstriction in the systemic circulation, resulting in unchanged or even slightly decreased systemic blood pressure [5]. However, a few weeks of exposure to hypobaric hypoxia can increase sympathetic activity, resulting in elevated heart rate and blood pressure [6]. Importantly, hypoxia accompanied by hypobaric conditions plays a key role in modulating the cardiovascular system.

Hypoxic preconditioning [7, 8] or brief ischemic preconditioning [9–12] under normobaric conditions has been well documented to provide cardioprotective effects against ischemia-reperfusion injury. Hong et al. demonstrated that whole-body hypoxic preconditioning significantly attenuated ischemia-reperfusion-induced myocardial injury 24 h after preconditioning in both hyper- and normotensive rats [7]. Hypoxia-inducible factor 1 alpha (HIF1*α*) is involved in these cardioprotective effects [7, 11, 13]. On the basis of previous studies, cardioprotective effects of hypobaric preconditioning via brief hypoxia may be expected. Previous studies have shown that chronic and/or intermittent exposure to hypobaric hypoxia has cardioprotective effects [14–16], and preservation of mitochondrial function and modulation of potassium channels sensitive to ATP have been shown in experimental models to be involved in these cardioprotective effects [17, 18].

Conversely, few studies have focused on the cardioprotective effects of acute and/or short-term hypobaric and hypoxic exposures. The aim of this study was to elucidate the effects of acute hypobaric and hypoxic exposures as preconditioning on myocardial ischemia-reperfusion injury in rats. We also investigated changes in HIF1*α* expression following acute hypobaric hypoxia exposures.

## 2. Material and Methods

All animal procedures and study protocols used in the present study were reviewed and approved by the Institutional Animal Care and Use of Committee of Sapporo Medical University (reference number 18-034) and adhered to the Guide for the Care and Use of Laboratory Animals (Institute for Laboratory Animal Research, 8th edition).

### 2.1. Hypobaric and Hypoxic Preconditioning

**Figure 1:**
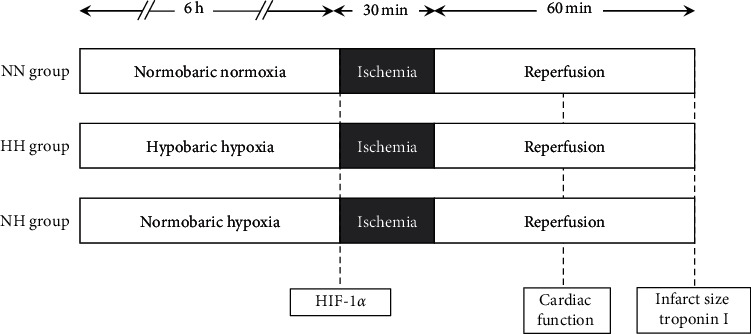
Experimental protocol. All rats were subjected to 30 min of ligation of the left anterior descending artery (LAD) followed by 60 min of reperfusion.

Male Wistar rats (250–300 g) were randomly assigned to three groups (*n* = 8 each): NN group, as a control group exposed to normobaric normoxia; HH group, as a group exposed to hypobaric hypoxia; and NH group, as a group exposed to normobaric hypoxia.  Figure 1 shows the experimental protocol. Rats in the HH group were exposed to hypobaric conditions of 60.8 kPa and hypoxic conditions of a 12.6% fraction of inspired oxygen (FiO_2_) in a hypobaric chamber for 6 h. We used a custom-built acrylic vacuum chamber (length, 70 cm; width, 25 cm; and height, 15 cm) pressurized to simulate an altitude of 4,600 m (60.8 kPa, simulating the upper limit of helicopter emergency medical services) or 0 m (101.3 kPa, sea level) using a vacuum pump (23 series, model 1023 V103; Gast Manufacturing, Benton Harbor, MI, USA) and controlled by a Honeywell controller (model UDC 3300; Honeywell, Morristown, NJ, USA) to maintain constant pressure. Oxygen concentrations were measured using a low-concentration oxygen monitor (JKO-O2LJD3; Ichinen Jiko, Tokyo, Japan). Rats in the NN group were exposed to normobaric conditions of 101.3 kPa, while rats in the NH group were exposed to an FiO_2_ of 12.6% using nitrogen under normobaric conditions for 6 h.

### 2.2. Ischemia-Reperfusion Injury In Vivo

After exposure to each condition for 6 h, myocardial ischemia-reperfusion injury was induced in all rats using the methods of Sawashita et al. [19]. Briefly, after rats were anesthetized intraperitoneally using a mixture of anesthetic agents (midazolam, 2 mg/kg; butorphanol, 2.5 mg/kg; and medetomidine, 0.15 mg/kg), the trachea was intubated with a 16-gauge cannula and the rat was mechanically ventilated using a volume-controlled mode with the tidal volume set to 1 mL/100 g body weight at a respiratory rate of 60 breaths/min (model 683 Small Animal Ventilator; Harvard Apparatus, Holliston, MA, USA). Systemic blood pressure, heart rate, and electrocardiograms were continuously monitored (AD Instruments, Sydney, Australia). Thoracotomy was performed and a 6-0 polyprolene loop was placed around the left anterior descending artery (LAD) to allow coronary artery occlusion and reperfusion. Myocardial ischemia was then induced by ligation of the LAD. All rats underwent 30 min of ischemia followed by 60 min of reperfusion. Myocardial infarction was confirmed on observing pallor of the left ventricle (LV) and ST segment elevation on electrocardiograms. Thirty minutes after reperfusion, fractional shortening (FS) and fractional area change (FAC) were measured echocardiographically. Sixty minutes after reperfusion, blood sampling was performed for analysis of cardiac troponin I concentrations. All collected blood samples were centrifuged at 1,500 × g at 4°C for 15 min to extract the plasma, which was subsequently stored at −80°C for enzyme-linked immunosorbent assay (ELISA).

### 2.3. Determination of Infarct Size and Cardiac Troponin

The LAD was then reoccluded and 4% Evans blue was injected to determine the “area at risk” (AAR) as previously described [19, 20]. The heart was excised under deep anesthesia, the LV was sliced at thickness of 2 mm and these slices were incubated in a 1% solution of 2,3,5-triphenyltetrazolium chloride dye for 15 min at 37°C and then fixed in 10% formalin for 20 min. AAR and infarct size were measured in a blinded manner using a planimetry method and Image J software (National Institutes of Health, Bethesda, MD, USA). AAR was expressed as a percentage of left ventricle area, and infarct size was expressed as a percentage of AAR.

Plasma concentrations of cardiac troponin I were measured using a commercially available ELISA kit (LSI Medience, Tokyo, Japan). Concentrations were measured spectrophotometrically using a standard 96-well plate reader at a wavelength of 450 nm (Sunrise™ reader; Tecan Group, Männedorf, Switzerland).

### 2.4. Protein Preparation and Immunoblotting

We examined the expression of HIF1*α*, which is known to play a crucial role in physiological changes under hypobaric and/or hypoxic conditions [21, 22]. To elucidate the effects of representative environments on HIF1*α* expression, we analyzed another 12 hearts from anesthetized rats (4 rats from each group) that had been exposed to each environmental condition for 6 h without inducing ischemia-reperfusion injury. Ventricular tissues were sampled 6 h after completing the exposure. Total protein was extracted with ice-cold buffer, and concentrations of protein were detected using a bicinchoninic acid Protein Assay Kit (Thermo Fisher Scientific, Waltham, MA, USA). Western blotting was performed as described previously [23]. Briefly, equal amounts of protein were separated by 7.5–12.5% sodium dodecyl sulfate-polyacrylamide gel electrophoresis and transferred to a nitrocellulose membrane (Bio-Rad Laboratories, Hercules, CA, USA). After blocking, membranes were incubated with antibodies directed against HIF1*α* (1 : 1000; Cell Signaling Technology, Beverly, MA, USA). All blots were analyzed in a blinded manner.

### 2.5. Statistical Analysis

On the basis of a previous study, a sample size of 6 rats in each group was needed to detect a 25% reduction in infarct size, which was considered appropriate (*α* = 0.05, 1 − *β* = 0.8, two-tailed) and clinically effective based on the G^*∗*^ Power 3.1 statistical power analysis program (Heinrich-Heine-University, Düsseldorf, Germany). All data were tested for normal distributions using the Shapiro–Wilk test. Data are presented as median and interquartile range (IQR) or mean and standard deviation. Cardiac function, infarct size/AAR, and levels of cardiac troponin and HIF1*α* expression were analyzed using one-way analysis of variance (ANOVA) followed by the Tukey's post hoc test. Hemodynamic status was analyzed by two-way repeated-measures ANOVA. All statistical analyses were performed using GraphPad Prism version 7.0 software (GraphPad Software, La Jolla, CA, USA). Statistical differences were considered significant for values of *p* < 0.05.

## 3. Results

No significant differences in blood pressure or heart rate were evident among groups at baseline and after exposure to acute hypobaric hypoxia. While mean blood pressure 15 min after ischemia and reperfusion were significantly lower compared to baseline in all groups, no differences in hemodynamics were evident among groups throughout the study (Table 1).

**Figure 2:**
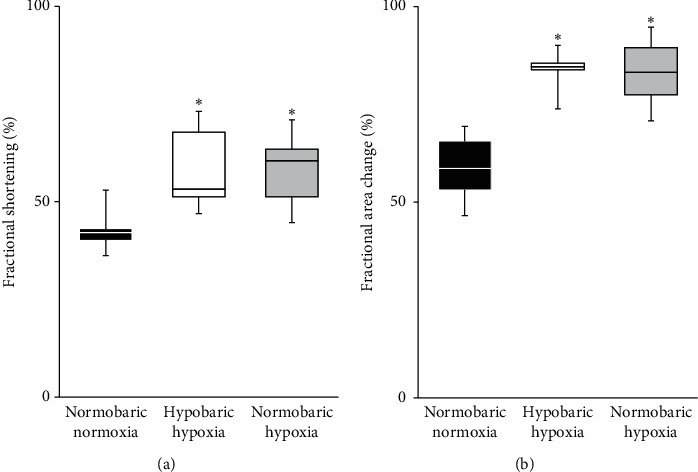
(a) Fractional shortening and (b) fractional area change in three groups. Data are shown as median and interquartile range (IQR), *n* = 8 rats per group. ^*∗*^*p* < 0.01 vs. normobaric normoxia group by one-way analysis of variance (ANOVA) followed by Tukey's post hoc test.

Figure 2 shows changes in FS (A) and FAC (B) at 30 min after reperfusion. Both FS and FAC were significantly higher in the HH and NH groups than in the NN group (median FS : NN group 42%, IQR 40–43%, HH group 53%, IQR 51–67%, *p* < 0.01 vs. NN group, NH group 60%, 51–63%, *p* < 0.01 vs. NN group; median FAC : NN group 58%, IQR 53–65%, HH group 84%, IQR 83–85%, *p* < 0.01 vs. NN group, NH group 83%, IQR 77–89%, *p* < 0.01 vs. NN group).

**Figure 3:**
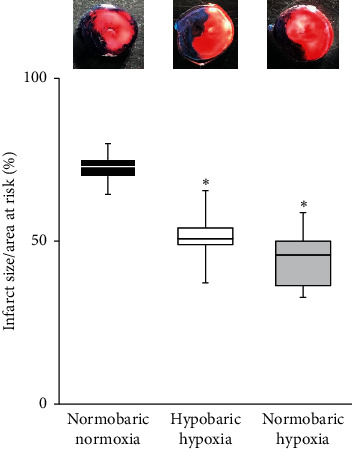
Infarct size/area at risk in the three groups. Representative TTC staining of heart slices after ischemia/reperfusion injury are presented for all groups. Infarct size is presented as a percentage of the area at risk. Data are shown as median and interquartile range (IQR), *n* = 8 rats per group. ^*∗*^*p* < 0.01 vs. normobaric normoxia group by one-way analysis of variance (ANOVA) followed by Tukey's post hoc test.

AAR was comparable among groups. Infarct size was significantly decreased in the HH and NH groups compared to the NN group. Median infarct size/AAR was significantly decreased in the HH and NH groups compared to that in the NN group (NN group 72%, IQR 69–75%; HH group 50%, IQR 48–54%, *p* < 0.01 vs. NN group; NH group 45%, IQR 36–50%, *p* < 0.01 vs. NN group) (Figure 3). No significant differences in cardiac function or infarct size were apparent between HH and NH groups.

**Figure 4:**
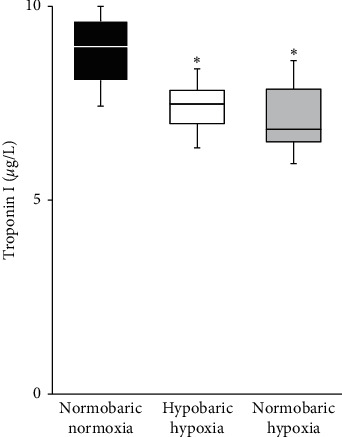
Plasma concentrations of troponin I in the three groups. Blood samples were collected from each group after 30 min of ischemia and 60 min of reperfusion. Data are shown as median and interquartile range (IQR), *n* = 8 rats per group. ^*∗*^*p* < 0.01 vs. normobaric normoxia group by one-way analysis of variance (ANOVA) followed by Tukey's post hoc test.

Plasma levels of cardiac troponin I were significantly lower in the HH and NH groups than in the NN group (Figure 4).

**Figure 5:**
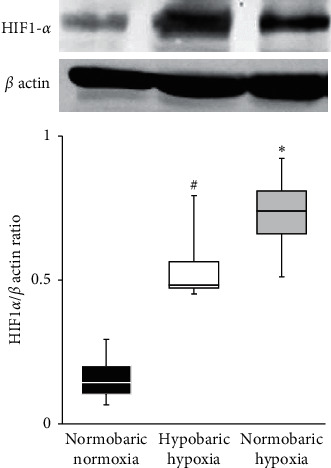
HIF1*α*/*β* actin ratio in the three groups in rat myocardium sampled from the left ventricle just after exposure to each condition. Representative immunoblots from all groups are presented. Arrows indicate positions of HIF1*α* and *β* actin bands. Data are shown as median and interquartile range (IQR), *n* = 4 rats per group. ^*#*^*p* < 0.05; ^*∗*^*p* < 0.01 vs. normobaric normoxia group by one-way analysis of variance (ANOVA) followed by Tukey's post hoc test.

Median HIF1*α* expression at 6 h after exposure to each environment was significantly higher in the HH and NH groups than in the NN group (NN group 0.14, IQR 0.10–0.20; HH group 0.48, IQR 0.47–0.56, *p* < 0.05 vs. NN group; and NH group 0.74, IQR 0.65–0.80, *p* < 0.01 vs. NN group) (Figure 5).

## 4. Discussion

This study revealed three main findings. First, acute hypobaric and hypoxic exposures exerted cardioprotective effects against ischemia-reperfusion injury in rats. Second, the cardioprotective effects from acute hypobaric hypoxic exposure were comparable to those from hypoxic exposure alone. Third, HIF1*α* might have contributed to these cardioprotective effects.

The effects of exposure to hypobaric and hypoxic environments on human health, including cardiovascular disease, have been studied for diverse populations residing at high altitude [14]. Hypobaric hypoxic exposure strongly affects the cardiovascular system. While short-term (a few hours) exposure induces systemic vasodilation and slightly decreased blood pressure, prolonged (a few weeks) exposure to hypoxia leads to increased sympathetic activity resulting in increases to heart rate, myocardial contractility, cardiac output, and blood pressure [5, 6]. Chronic exposure to hypobaric hypoxic environments can induce remodeling of the cardiovascular system, erythrocytosis, neurological symptoms, and pulmonary hypertension [24]. Conversely, recent evidence has shown benefits from high-altitude environments on cardiovascular disease [25, 26]. Interestingly, postoperative outcomes after cardiac surgery among patients living at high altitude have been shown to be better than those among patients living at low altitude [27, 28]. In animal studies, chronic and/or intermittent exposure to a hypobaric hypoxic environment can attenuate ischemia-induced arrhythmias, improve the efficiency of ATP generation [17], decrease age-related remodeling [18], and bring about improvements in myocardial capillaries [29], cardiac function, and mitochondrial function [30, 31]. Beneficial effects of chronic and/or intermittent exposures to hypobaric hypoxic environments on the cardiovascular system have thus been identified. Conversely, the effects of acute hypobaric hypoxic exposure on the cardiovascular system have not been fully elucidated, although the numbers of people exposed to acute high-altitude environments during activities such as mountain climbing and air travel have increased. Although one study investigated the effects of acute hypobaric hypoxic conditions for 6 h on acute skeletal muscle edema after ischemia-reperfusion injury [32], no studies have focused on the effects of acute exposure to high altitude on myocardial ischemia-reperfusion injury. The present study showed that preconditioning with acute hypobaric hypoxia resulted in cardioprotective effects against myocardial ischemia-reperfusion injury.

Brief hypoxic (ischemic) exposure is well documented to exert cardioprotective effects against ischemia-reperfusion injury [7–12]. The cardioprotective effects presented in the present study might be induced simply with hypoxic conditions alone. To clarify the contribution of hypobaria to any cardioprotective effects, we compared the effects between hypobaric hypoxic and normobaric hypoxic conditions. Ours results suggested no differences in the cardioprotective effects between groups. At the very least, hypobaric conditions did not interfere with the cardioprotective effects induced by hypoxic preconditioning alone. We have tried to conduct a study comparing the cardioprotective effects between hypobaric hypoxic conditions and hypobaric normoxic conditions in order to clarify the effects of hypobaria alone. However, establishing a hypobaric normoxic condition using oxygen loading was difficult in the hypobaric chamber utilized in our study due to the instability of oxygen concentration and difficulties in maintaining a stable barometric pressure.

HIF1*α* contributed to the cardioprotective effects identified in the present study. HIF is known to play a crucial role in transcriptional responses to changes under hypoxic conditions [33]. HIF1*α* can induce tissue preservation in experimental models of myocardial infarction [34] and stroke [35]. HIF1*α* plays a pivotal role in the cardioprotective effects of ischemic preconditioning against ischemia-reperfusion injury, via production of mitochondrial reactive oxygen species [13]. Under hypobaric hypoxic conditions, HIF1*α* expression depends on the degrees of hypobaria and hypoxia and duration of exposure according to studies of the brain [36, 37]. Although chronic exposure to hypobaric hypoxic conditions modulates HIF1*α* expression [14], the effects of acute hypobaric hypoxic conditions have not been clarified. Our results showed that combined hypobaric hypoxic exposure activated HIF1*α* expression, which might be involved in the cardioprotective effects.

Several limitations need to be considered when interpreting the results of this study. First, our animal experiments were performed using an established model of ischemia-reperfusion injury in rats. Our results thus are not necessarily indicative of the mechanisms involved or the results that would be obtained in humans. While we identified beneficial effects of hypobaric hypoxia preconditioning before ischemia-reperfusion, predicting myocardial ischemia events is difficult in clinical settings. In addition, to apply this cardioprotective effect to humans in clinical settings, establishment of a medical hypobaric hypoxic environment would be required. Further accumulation of basic and clinical evidence is thus desirable to enhance the efficacy of hypobaric preconditioning and identify the clinical relevance. Second, we could not determine the effects of hypobaric conditions alone (i.e., under normoxic conditions) on cardioprotective effects against ischemia-reperfusion injury, because our hypobaric chamber did not allow stable maintenance of barometric pressures under oxygen loading. Determination of the cardioprotective effects of hypobaric conditions alone merits further investigation. Third, the hypobaric hypoxia applied in this study was limited to hypobaric conditions of 60.8 kPa and hypoxic conditions of an FiO_2_ of 12.6% in a hypobaric chamber for 6 h. We set this condition to simulate an altitude of 4,600 m, as the upper limit of helicopter emergency medical services. Differential hypobaric hypoxic conditions might lead to results differing from those obtained in this study.

Finally, details of the mechanisms by which HIF1*α* mediates any cardioprotective effects could not be elucidated from the present study. Several biomarkers, including prosurvival kinases (e.g., Akt, mitogen-activated protein kinase, and nitric oxide synthase) [7–12] and mitochondrial bioenergetics [20], would need to be analyzed to elucidate details of the interactions between HIF1*α* and cardioprotective effects in hypobaric hypoxic preconditioning.

## 5. Conclusion

The present study demonstrated that acute hypobaric hypoxia can induce cardioprotective effects against ischemia-reperfusion injury in rats. HIF1*α* may contribute to these cardioprotective effects.

## Figures and Tables

**Table 1:** Changes in mean blood pressure and heart rates.

Mean blood pressure (mmHg)	Baseline	15 min after ischemia	Reperfusion	
			30 min	60 min
NN group	131.9 ± 12.1	104.1 ± 17.8^*∗*^	81.0 ± 13.8^*∗*^	74.8 ± 18.1^*∗*^
HH group	126.1 ± 18.6	97.1 ± 19.9^*∗*^	85.5 ± 20.8^*∗*^	87.8 ± 19.7^*∗*^
NH group	139.0 ± 20.4	105.0 ± 14.6^*∗*^	85.8 ± 9.6^*∗*^	76.5 ± 11.6^*∗*^
Hear rate (bpm)				
NN group	275.6 ± 30.9	311.5 ± 40.6	278.5 ± 30.1	292.0 ± 43.5
HH group	269.5 ± 37.3	296.7 ± 43.7	288.4 ± 34.3	312.2 ± 41.8
NH group	298.1 ± 23.6	312.3 ± 30.2	283.2 ± 23.2	308.6 ± 45.5

Data are shown as mean ± standard deviation (SD). NN: normobaric normoxia; HH: hypobaric hypoxia; and NH: normobaric hypoxia. ^*∗*^*p* < 0.05 vs. baseline in each group, *n* = 8, as assessed by using two-way ANOVA followed by Tukey's post hoc test.

## Data Availability

The data that support the findings of this study are available from the corresponding author, N.H., upon reasonable request.
